# Phenylmethimazole abrogates diet-induced inflammation, glucose intolerance and NAFLD

**DOI:** 10.1530/JOE-18-0078

**Published:** 2018-04-17

**Authors:** Ashley Patton, Tyler Church, Caroline Wilson, Jean Thuma, Douglas J Goetz, Darlene E Berryman, Edward O List, Frank Schwartz, Kelly D McCall

**Affiliations:** 1 Department of Specialty MedicineHeritage College of Osteopathic Medicine, Ohio University, Athens, Ohio, USA; 2 Diabetes Institute Ohio University, Athens, Ohio, USA; 3 Department of Biological SciencesOhio University, Athens, Ohio, USA; 4 Molecular & Cellular Biology ProgramCollege of Arts and Sciences, Ohio University, Athens, Ohio, USA; 5 Department of Chemical and Biomolecular EngineeringRuss College of Engineering and Technology, Ohio University, Athens, Ohio, USA; 6 Biomedical Engineering ProgramOhio University, Athens, Ohio, USA; 7 Department of Biomedical SciencesOhio University, Athens, Ohio, USA; 8 The Edison Biotechnology InstituteOhio University, Athens, Ohio, USA

**Keywords:** phenylmethimazole (C10), NAFLD, glucose intolerance, type 2 diabetes, inflammation

## Abstract

Nonalcoholic fatty liver disease (NAFLD) is the hepatic manifestation of both metabolic and inflammatory diseases and has become the leading chronic liver disease worldwide. High-fat (HF) diets promote an increased uptake and storage of free fatty acids (FFAs) and triglycerides (TGs) in hepatocytes, which initiates steatosis and induces lipotoxicity, inflammation and insulin resistance. Activation and signaling of Toll-like receptor 4 (TLR4) by FFAs induces inflammation evident in NAFLD and insulin resistance. Currently, there are no effective treatments to specifically target inflammation associated with this disease. We have established the efficacy of phenylmethimazole (C10) to prevent lipopolysaccharide and palmitate-induced TLR4 signaling. Because TLR4 is a key mediator in pro-inflammatory responses, it is a potential therapeutic target for NAFLD. Here, we show that treatment with C10 inhibits HF diet-induced inflammation in both liver and mesenteric adipose tissue measured by a decrease in mRNA levels of pro-inflammatory cytokines. Additionally, C10 treatment improves glucose tolerance and hepatic steatosis despite the development of obesity due to HF diet feeding. Administration of C10 after 16 weeks of HF diet feeding reversed glucose intolerance, hepatic inflammation, and improved hepatic steatosis. Thus, our findings establish C10 as a potential therapeutic for the treatment of NAFLD.

## Introduction

Obesity is the single most important risk factor for the development of nonalcoholic fatty liver disease (NAFLD), which is the most prevalent liver disease in the western hemisphere (Lazo & Clark 2008, Bellentani *et al*. 2010). NAFLD, the hepatic manifestation of metabolic syndrome (Yki-Jarvinen 2014), is linked to visceral obesity (a systemic pro-inflammatory state), dyslipidemia, insulin resistance and type 2 diabetes mellitus (T2DM) (Zelber-Sagi *et al*. 2006). Specifically, NAFLD is a disease of excess fat accumulation in the liver of individuals with no history of alcohol abuse, which can range from benign steatosis to advanced steatohepatitis (NASH) and cirrhosis (El-Serag & Kanwal 2014). NASH is associated with increased mortality not only from vascular disease but also from complications of cirrhosis and hepatocellular cancer. Thus, targeting hepatocellular inflammation is expected to significantly prevent the progression of the disease and reduce mortality in patients with NAFLD (Younossi *et al*. 2011).

High-fat (HF) diets promote weight gain leading to an increase in adipose tissue mass (i.e. obesity). Simultaneously, these HF diets cause an increase in levels of circulating free fatty acids (FFAs) and triglycerides (TGs) that deposit in adipose tissue as well as in the liver and skeletal muscle (i.e. ectopic fat deposition) (Day 2002, Dowman *et al*. 2010). Ectopic fat deposition in the liver is the hallmark of hepatic steatosis, which is the earliest stage of NAFLD and is associated with the development of insulin resistance (Day & James 1998, Surwit *et al*. 1988, Xu *et al*. 2003, Cani *et al*. 2007). The ectopically deposited FFAs and TGs observed in steatosis induce a local, low-grade hepatic inflammation (Weisberg *et al*. 2003, Glass & Olefsky 2012) and is otherwise a benign disease at this stage (Jia *et al*. 2014, Sawada *et al*. 2014). Unfortunately, steatosis often leads to the development of NASH, which is characterized by immune cell infiltrate, hepatocyte injury and/or fibrosis.

Pathological activation and signaling of toll-like receptor 4 (TLR4) by non-immune ligands (including FFAs) and immune ligands (including gut-derived lipopolysaccharide or LPS) contribute to the inflammation present in NAFLD (Lu *et al*. 2008, Broering *et al*. 2011). TLR4 signaling is mediated via two intracellular pathways involving the myeloid differentiation primary response 88 (MyD88) or adaptor proteins translocation-associated membrane protein 1/TIR-domain-containing adapter-inducing interferon-β (TRIF) (Holland *et al*. 2011). In the MyD88-dependent pathway, MyD88 signals the activation of IL-1 receptor-associated kinases (IRAK4/IRAK1) and TNF receptor-associated factor (TRAF6) to activate nuclear factor κB (NFKB) and activated protein 1 (AP1) leading to the induction of pro-inflammatory cytokines (IL6, TNFA) (Paik *et al*. 2003, Takeda *et al*. 2003, Shi *et al*. 2006, Miura *et al*. 2013, O’Neill *et al*. 2013). In the MyD88-independent pathway, TLR4 recruits TRAF3 and receptor-interacting protein 1 (RIP1) by TRIF/toll-like receptor adaptor molecule 1 (TICAM1) to promote the downstream activation of TANK-binding kinase 1 (TBK1) and inhibitor κB kinase ε (IKKE) (Seki *et al*. 2001, Akira *et al*. 2006, Schneider *et al*. 2006). This molecular complex (TRIF/TICAM1/TRAF3/RIP1/TBK1/IKKE) phosphorylates interferon regulatory factor 3 (IRF3) (Fitzgerald *et al*. 2003). Following phosphorylation, IRF3 homodimerizes and translocates to the nucleus where it induces type 1 interferon expression (e.g. IFNB (Fitzgerald *et al*. 2003, Hemmi *et al*. 2004)). Indirectly, IRF3 interacts with NFKB and AP1 to induce IL6 and TNFA expression.

Activation of TLR4-mediated inflammation also exacerbates hepatic lipid accumulation, although the exact mechanism is still unknown. Mice deficient in TLR4 demonstrate HF diet-induced weight gain but are protected against inflammation, hepatic steatosis and insulin resistance (Shi *et al*. 2006, Poggi *et al*. 2007, Suganami *et al*. 2007, Tsukumo *et al*. 2007, Davis *et al*. 2008, Spruss *et al*. 2009, Pierre *et al*. 2013, Jia *et al*. 2014, Ferreira *et al*. 2015). Liver-specific TLR4-knockout (TLR4^LKO^) mice become obese when placed on a HF diet but remain insulin sensitive and are protected from the development of steatosis (Jia *et al*. 2014). The attenuation of steatosis and insulin resistance is most likely due to reduced pro-inflammatory gene expression in liver and adipose tissue of both global and liver-specific TLR4-deficient mice (Jia *et al*. 2014).

Even with the acknowledged epidemic of obesity and associated NAFLD, there is an overwhelming failure (1) to clinically recognize the disease in the early stages due to the lack of specific diagnostic indicators or (2) to initiate treatment as there are no effective medications which specifically attenuate the early systemic inflammatory processes of NAFLD. This leaves patients and physicians only with long-term weight loss through diet to treat NAFLD, which is effective but very difficult to sustain (Gelli *et al*. 2017), or bariatric surgery, which can be very effective at reducing hepatic fat content (Hannah & Harrison 2016, Schwenger *et al*. 2018) but can have significant associated complications (Chang *et al*. 2017). In light of this and studies suggesting a direct involvement of TLR4-mediated inflammation in the development of HF diet-induced hepatic steatosis and insulin resistance, there is a concerted effort directed at developing therapeutics targeting TLR4 signaling. We have developed a library of small-molecule inhibitors of inflammation that potently block TLR signaling, including FFA- and gut-derived LPS-induced TLR4 signaling (Harii *et al*. 2005, McCall *et al*. 2007, 2010, 2013, Schwartz *et al*. 2009, Deosarkar *et al*. 2014). Our lead compound, phenylmethimazole (C10), is a derivative of methimazole that inhibits inflammation resulting from TLR3 and TLR4 signaling in both immune and non-immune cells by blocking homodimerization of IRF3 and thus blocking its nuclear translocation and transcriptional activation activity (Courreges *et al*. 2012). Thus, we hypothesized that C10 will prevent and/or reverse HF diet-induced hepatic and adipose tissue inflammation as well as hepatic steatosis and glucose intolerance in a diet-induced obesity (DIO) mouse model.

## Materials and methods

### Phenylmethimazole (C10) solutions

Phenylmethimazole (C10) (Concord Biosciences, Cleveland, OH, USA) was prepared as a 200 mM stock solution in 100% (v/v) DMSO (Sigma-Aldrich) and further diluted to achieve the working concentration indicated in individual experiments.

### Cell culture

Mouse hepatocyte cell line, AML-12 (ATCC), was cultured in DMEM-F12 with 0.005 mg/mL insulin, 0.005 mg/mL transferrin, 5 ng/mL selenium and 40 ng/mL dexamethasone, 10% (v/v) fetal bovine serum (Gibco) and 1% (v/v) penicillin/streptomycin. Human hepatocellular carcinoma cell line, HepG2 (ATCC) was cultured in DMEM, 10% (v/v) fetal bovine serum (Gibco) and 1% (v/v) penicillin/streptomycin (Gibco). Both cell lines were grown at 37°C with 5% CO_2_. A working solution of C10 (100 µM) was prepared in 0.25% DMSO (Sigma-Aldrich). For palmitate and LPS treatments, cells (cell passages <20) were incubated with 750 µM palmitate (Sigma-Aldrich) solution conjugated to FFA-free BSA (Sigma-Aldrich), 2% in serum-free culture media or complete culture media containing 10 ng/mL LPS (Sigma-Aldrich).

### Mice and experimental design

This work was conducted with approval from the Ohio University Institutional Animal Care and Use Committee in accord with accepted standards of humane animal care.

### Experimental procedures

Six-week-old C57BL/6J male mice were purchased from Jackson Labs and housed 4 per cage in an environment controlled for temperature (18–22°C) and humidity on a 14:10-h light/darkness cycle. Mice were allowed to acclimate for 1 week prior to diet placement and C10 and control treatments.

#### Prevention study

Prior to the start of the experiment, mice were randomly assigned to a treatment group: low-fat (LF) diet ((#D12450B, Research Diets Inc., New Brunswick, NJ, USA) (10% fat, 20% protein, 70% carbohydrate)) sham injection, HF diet ((#D12492, Research Diets Inc) (60% fat, 20% protein, 20% carbohydrate)) sham injection, HF diet + DMSO, HF diet + 1 mg/kg C10 in 10% DMSO and PBS. Mice received intraperitoneal (IP) injections once daily for 18 weeks. Weights were recorded weekly. Body composition (%fat, %fluid and %lean measurements) was obtained using the Bruker Minispec Whole Body Composition Analyzer (Billerica, MA, USA). An intraperitoneal glucose tolerance test (IPGTT) was performed on mice after 13 weeks on their respective diets and initiation of treatment.

#### Reversal study

Prior to the start of the experiment, mice were randomly assigned to a diet group: LF diet group or HF diet group. After 16 weeks on their respective diet, an IPGTT was performed to evaluate glucose tolerance in each mouse. Any mouse in the LF diet group that was glucose intolerant and any mouse in the HF diet group that were glucose tolerant were removed from the study. Inclusion/exclusion criteria were as follows: If the IPGTT curve for a HF diet-fed mouse was identical or very similar to that of the LF diet-fed group, it was excluded. Similarly, if the IPGTT curve for a LF diet-fed mouse was identical or very similar to that of the HF diet-fed group, it was excluded. One week following the IPGTTs (i.e. after 17 weeks on respective diets), mice fed the HF diet were randomly assigned to a treatment group; HF diet + sham injection, HF diet + DMSO, HF diet + 1 mg/kg C10 in 10% DMSO and PBS. Mice received once daily IP injections for 14 weeks. Weights were recorded weekly. Body composition was obtained as described earlier. Another IPGTT was performed on mice after 12 weeks of C10 or control treatments.

### Intraperitoneal glucose tolerance tests (IPGTTs)

Intraperitoneal glucose tolerance tests (IPGTTs) were performed on 12-h fasted mice. Body weight and blood glucose (Freestyle Freedom Blood Glucose monitoring System, Abbott Laboratories) was measured prior to IP injection of glucose (Sigma-Aldrich) (1–2 g/kg body weight). Subsequent blood glucose measurements were performed at time 0 and at 20/30, 60, 90, 120 and 180 min post IP injection of the glucose bolus.

### Histological analysis

For microscopic examination of liver morphology and steatosis, liver tissue was fixed in 10% buffered formalin for 12–24 h. Formalin-fixed tissues were dehydrated in ethanol and embedded in paraffin for hematoxylin and eosin staining. Liver sections for histological staining were cut to 5 µm. Tissue preparation for histological analysis was performed by Ohio University Heritage College of Osteopathic Medicine Histological Core Services.

### Hepatic TG quantification

Hepatic TG content was evaluated using a protocol based on the Salmon and Flatt method of lipid saponification (Salmon & Flatt 1985, List *et al*. 2009). Glycerol concentration was plotted against absorbance. The concentration of glycerol (mg glycerol/g tissue) was calculated by multiplying the determined concentration from the equation of the graph by the dilution factor and the number 5.31. The number (5.31) was used to correct the conversion of glycerol to TG by units of glycerol (mg/dL) to units of TG (mmol/L) and to mg/g tissue. TG content in AML-12 cells was presented as a ratio of total protein determined by BCA.

### Serum TG and total cholesterol

Serum TG and total cholesterol were measured using blood collected at the experimental endpoint from non-fasted mice. The commercially available colorimetric Triglyceride Quantification Assay Kit (Abcam, cat #ab65336) was performed according to the manufacturer protocol to quantify serum TG. The commercially available colorimetric Cholesterol/Cholesteryl Ester Quantitation Assay Kit (Abcam, cat #ab65359) was used according to the manufacturer protocol to measure serum total cholesterol.

### Quantitative real time-PCR analysis

Total RNA was isolated from cells in culture or frozen liver and mesenteric adipose tissue using TRIzol reagent (Invitrogen, Thermo Fischer Scientific). Preparation of cDNA was achieved using the high-capacity cDNA reverse transcription kit with RNase Inhibitor (Applied Biosystems, Thermo Fischer Scientific). TaqMan (Applied Biosystems, Thermo Fischer Scientific) and SYBR Green (Bio-Rad) biochemistries was used to perform qRT-PCR to quantify gene expression according to the manufacturer’s protocol. Murine TaqMan gene expression assays used include: *Ifnb* (Mm00439552_s1), *Adgre1* (*Emr1;F4/80*) (Mm00802529_m1), *Il6* (Mm00446190_m1) and *Gapdh* (Mm99999915_g1) as the housekeeping gene. Mouse *Tnfa* primers were as follows: sense primer, 5′-Cgg TCC CCA AAg GGA TgA g-3′; antisense primer, 5′ CCT TgA AgA gAA CCT ggg AgT A-3′. Human TaqMan gene expression assays used include: *TNFA* (Hs00174128_m1), *IFNB1* (Hs01077958_s1) and *GAPDH* (Hs02786624_g1) as the housekeeping genes.

### Statistical analysis

Statistical analysis was performed using GraphPad Prism 7 for Mac. Statistical differences were determined using a one-way or two-way ANOVA followed by a Tukey–Kramer or Bonferroni test for *post hoc* comparison.

## Results

### C10 prevents FFA- and LPS-induced inflammation in both murine and human hepatocytes in culture and TG accumulation in murine hepatocytes

**Figure 1:**
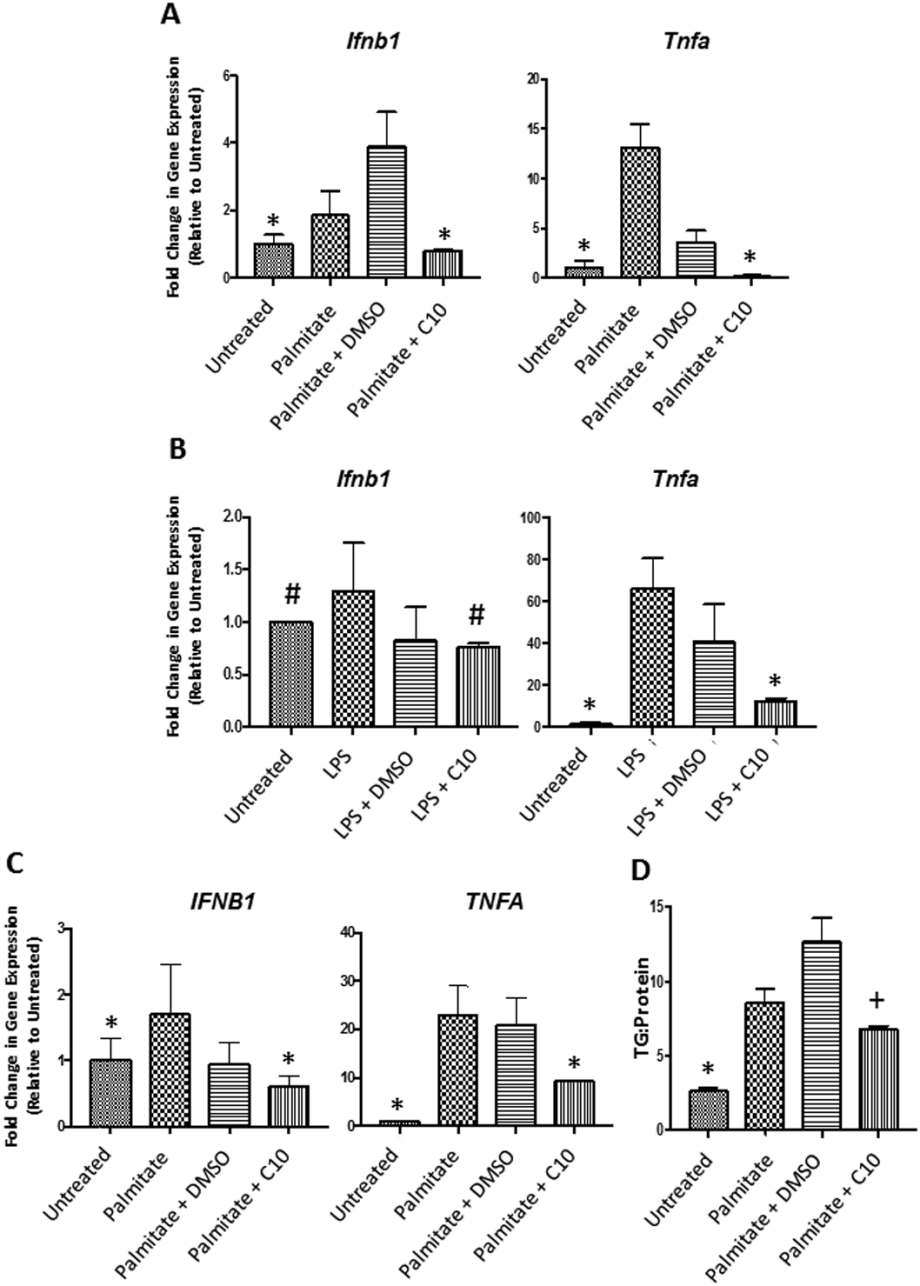
C10 inhibits hepatic inflammation in addition to triglyceride accumulation in cell culture. AML-12 and HepG2 cells were treated with 100 µM C10 or DMSO (control) to determine if C10 could prevent hepatic inflammation in the presence of 0.75 mM palmitate or 10 ng/mL LPS. Treatment with C10 prevented palmitate- (A) and LPS (B)-induced pro-inflammatory cytokine (*Ifnb1* and *Tnfa*) expression. Inhibition of palmitate-induced pro-inflammatory cytokine expression was also observed in HepG2 cells (C). Treatment with C10 prevented palmitate-induced triglyceride accumulation in AML-12 cells (D). Bars indicate mean + s.e.m. Significance was determined using a one-way ANOVA followed by Tukey’s *post hoc* analysis for multiple comparison; *P < 0.05 between Untreated and Palmitate + C10 treated groups compared to both Palmitate and Palmitate + DMSO groups (A, C and D) or *P* < 0.05 between Untreated and LPS + C10 treated groups compared to LPS and LPS + DMSO groups (B). ^#^Different from LPS,* P* < 0.05. ^+^Different from Palmitate and Palmitate + DMSO,* P* < 0.05 (D).

In previous studies, we have shown that C10 prevents palmitate- and LPS-induced pro-inflammatory cytokine expression in murine macrophages (RAW264.7 cells) and differentiated 3T3-L1 adipocytes by inhibiting TLR4 signaling, specifically by blocking transcriptional activity of IRF3 (McCall *et al*. 2010). In HFD-induced NAFLD, TLR4 expressed in hepatocytes is activated by both FFAs and LPS (Matsumura *et al*. 2000, Reyna *et al*. 2008). Stimulation of the MyD88-dependent pathway leads to pro-inflammatory cytokine expression, specifically *Tnfa* and *Il6*, while activation of the MyD88-independent TLR4 pathway leads to direct upregulation of type 1 interferons (*Ifnb1*) and indirect upregulation of *Tnfa* (Paik *et al*. 2003, Takeda *et al*. 2003, Shi *et al*. 2006, Miura *et al*. 2013, O’Neill *et al*. 2013). As indicated in Fig. 1A and B, treatment with palmitate and LPS leads to the upregulation of *Ifnb1* and *Tnfa* in murine hepatocytes (AML-12 cells) compared to the untreated control groups and C10 prevents LPS- and palmitate-induced upregulation of *Ifnb1* and *Tnfa*. Treatment with C10 also prevents palmitate-induced pro-inflammatory cytokine expression in HepG2 cells, a human hepatocellular carcinoma cell line (Fig. 1C). The solvent control, DMSO, is known to exhibit anti-inflammatory effects by repressing pro-inflammatory cytokine production (Elisia *et al*. 2016). Although DMSO had some anti-inflammatory activity, we show that C10 has a greater anti-inflammatory effect by inhibiting pro-inflammatory cytokine production when compared to the palmitate- and LPS-stimulated DMSO groups.

Inflammation is associated with enhanced hepatic *de novo* lipogenesis and TG accumulation (Feingold & Grunfeld 1987, Grunfeld *et al*. 1988, 1991, Feingold *et al*. 1990, 1992). Exogenous *Tnfa* in mice and rats has caused increased TG production and storage in the liver (Feingold & Grunfeld 1987, Feingold *et al*. 1990). In addition to preventing FFA- and LPS-induced pro-inflammatory cytokine expression *in vitro*, C10 also reduced palmitate-mediated accumulation of TG in mouse hepatocytes (AML-12 cells) (Fig. 1D).

### C10 does not affect weight gain, body composition or adipose weight in HF diet-fed C57BL/6J male mice

**Figure 2:**
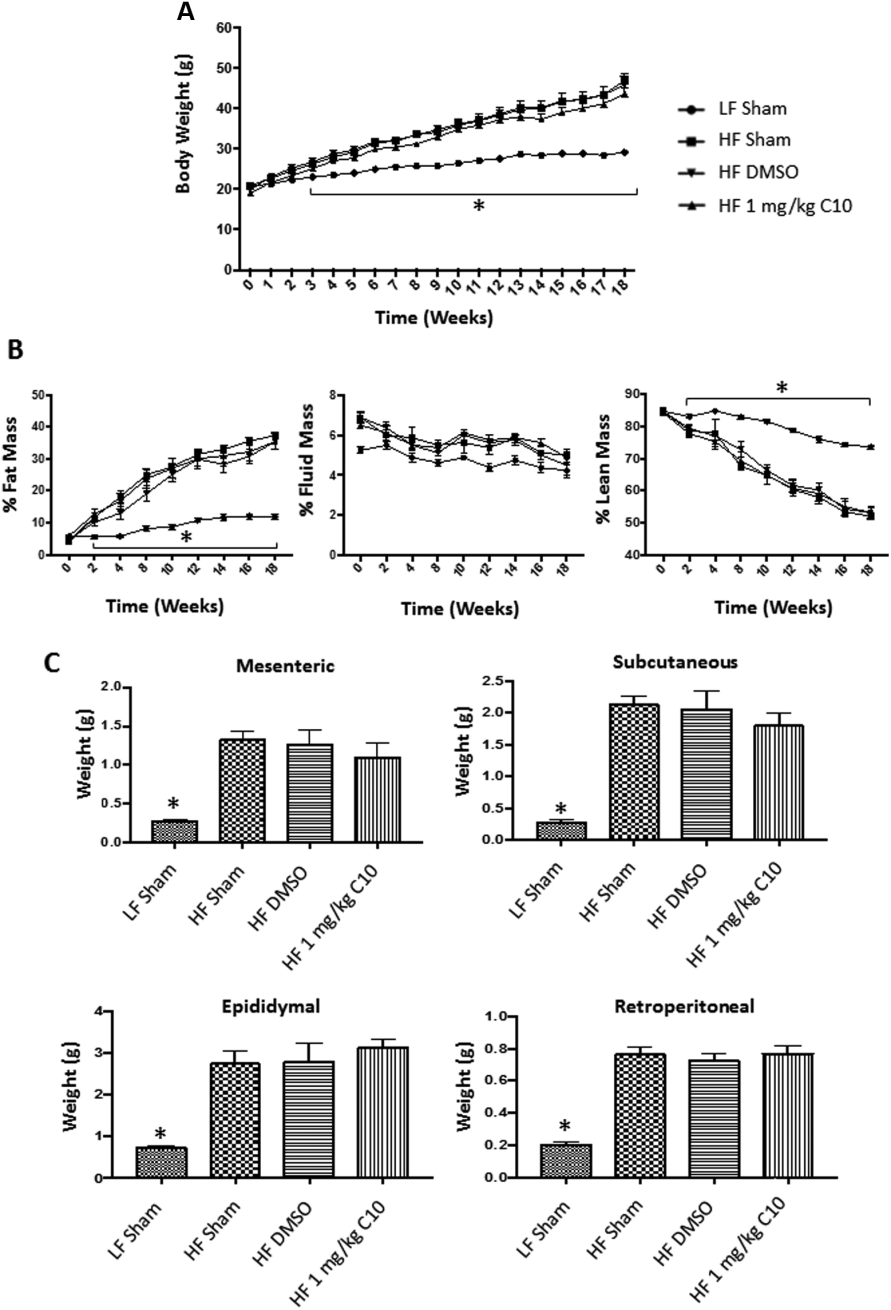
C10 does not prevent weight gain or an increase in fat mass due to HF-feeding. Seven-week old C57BL/6J male mice were fed either LF or HF diet and treated once daily with sham, DMSO, or C10 intraperitoneal injection for 18 weeks. Total body weights were measured weekly and body composition was measured every 2 weeks for the duration of the study. Adipose (mesenteric, subcutaneous, epididymal, and retroperitoneal) tissue weight was measured after tissue harvest at 18 weeks. (A) HF diet feeding promoted a marked increase in body weight when compared to LF-fed mice. (B) Additionally, % Fat mass was increased in HF-fed mice when compared to LF-fed controls. Percent Lean Mass was increased in LF-fed mice when compared to HF-fed groups. Percent Fluid Mass was no different between LF- and HF-fed mice. (C) HF-fed mice displayed increased adipose tissue weights after 18 weeks on HF diet when compared to LF-fed mice. Data points on line graphs (A and B) indicate mean and error bars indicate +/− s.e.m. and bars on bar graphs (C) indicate mean + s.e.m. Significance was determined using ANOVA followed by Tukey’s *post hoc* analysis for multiple comparison; *Different from HF-fed groups; *P* < 0.05, *n* *=* 8.

It is already known that TLR4-deficient mice develop obesity when fed a HF diet. Thus, we were interested in the effect of C10 treatment on weight and body composition in a HF diet-induced model of obesity (DIO model). Seven-week-old C57BL6/J male mice were fed either a LF diet (10% fat) or HF diet (60% fat). Mice were dosed once daily with intraperitoneal (IP) sham injections, IP injections of DMSO (vehicle) or IP injections of 1 mg/kg C10 for 18 weeks. During the 18-week study (Prevention Study), mice were evaluated for the development of obesity by measurement of body weight and body composition. A HF diet challenge resulted in significantly more weight gain compared to the LF-fed mice (Fig. 2A). Body composition revealed increased fat mass as a percentage of total body weight in the HF-fed mice when compared to the LF-fed mice (Fig. 2B). Obesity was also assessed by adipose tissue weight. At the end of the 18-week study, HF-fed mice had increased adipose tissue weight in mesenteric, subcutaneous, epididymal and retroperitoneal depots when compared to the LF sham group (Fig. 2C). C10 and DMSO treatment had no significant effect on weight, body composition or adipose tissue weight of HF-fed mice (Fig. 2).

### C10 blocks hepatic TG deposition in HF diet-fed C57BL/6J male mice

**Figure 3:**
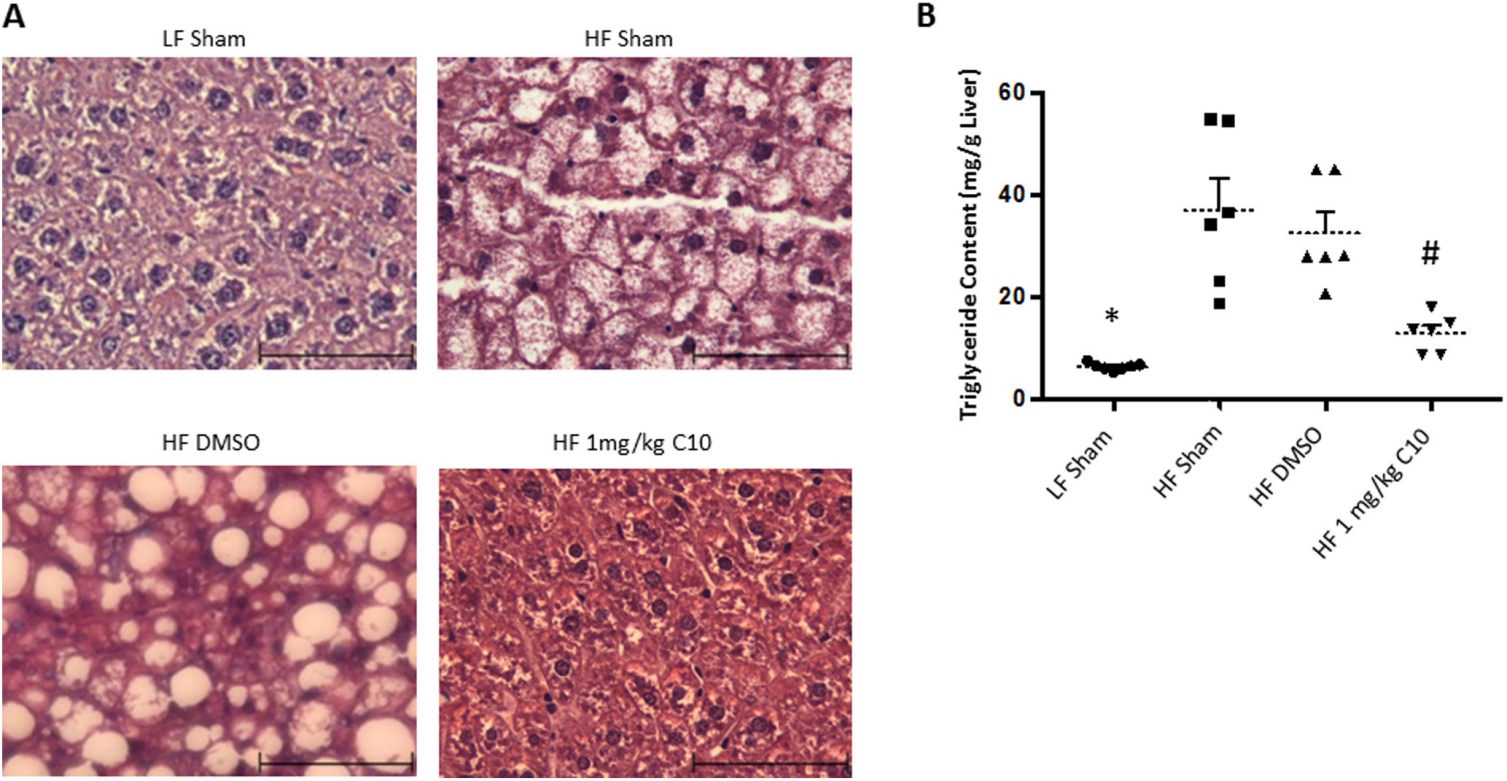
C10 prevents HF diet-induced hepatic steatosis. Hematoxylin and eosin staining was performed on liver tissue sections prepared after 18 weeks of HF diet feeding. Liver triglyceride content was determined by biochemical analysis (A) Histological examination revealed that C10 prevents hepatic lipid accumulation. All images in (A) were taken at 400× magnification. Scale bar, 40 µm. (B) Treatment with C10 decreased hepatic triglyceride content when compared to HF-fed sham and DMSO groups but had no effect on serum triglyceride levels. Dotted lines represent the mean and error bars indicate + s.e.m. Significance was determined using ANOVA followed by Tukey’s *post hoc* analysis for multiple comparison; *Different from HF-fed groups; *P* < 0.05. ^#^Different from HF sham and HF DMSO groups; *P* < 0.05.

**Figure 4:**
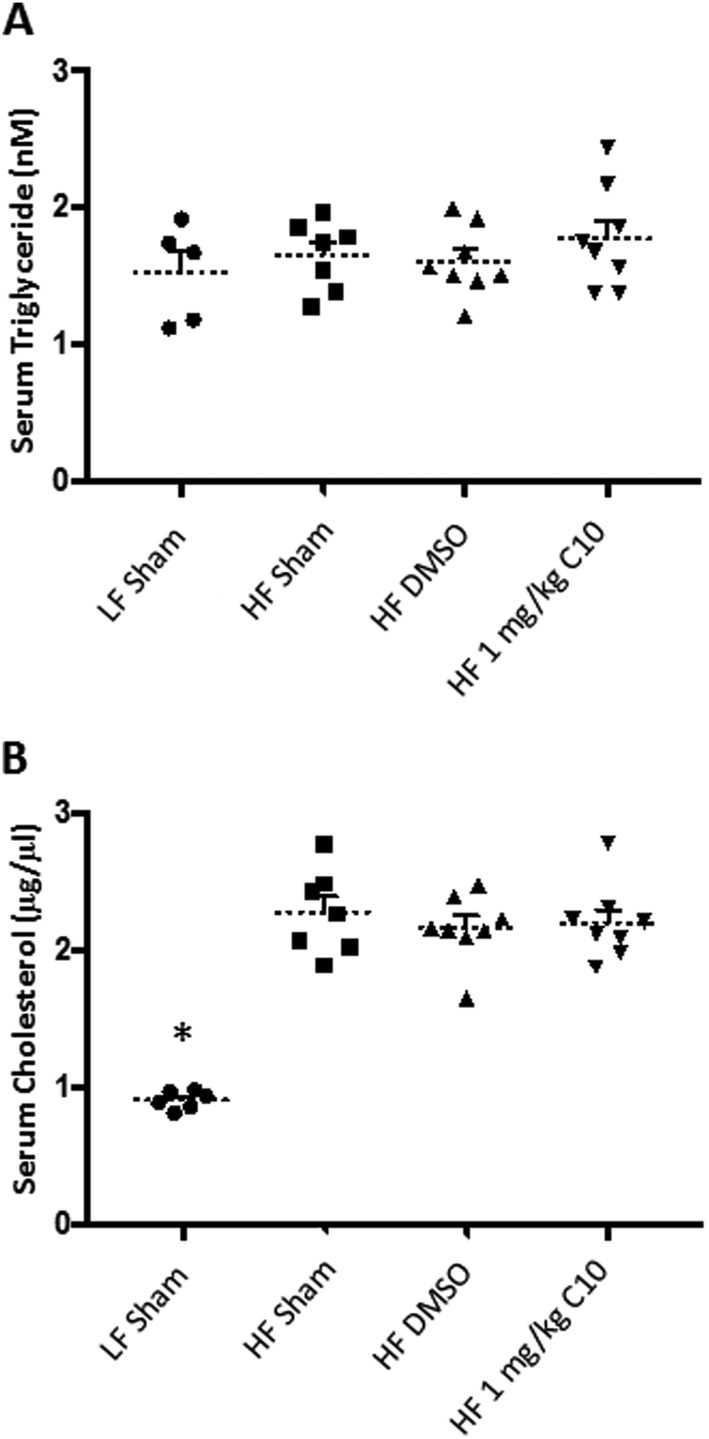
Total serum cholesterol is elevated in HF diet-fed mice, but serum triglyceride is unchanged. Total serum triglyceride and cholesterol were measured from non-fasted mice after 16 days of diet challenge and/or C10 treatment using commercially available colorimetric assays. (A) Serum triglyceride levels were unaffected by HF diet feeding and C10 treatment. (B) Total serum cholesterol was elevated in HF-fed mice when compared to LF sham controls. Dotted lines represent the mean and error bars indicate + s.e.m. Significance was determined using ANOVA followed by Tukey’s *post hoc* analysis for multiple comparison; *Different from HF-fed groups; *P* < 0.05, *n* = 6–8.

Our *in vitro* experiments demonstrated that C10 prevented palmitate-induced TG accumulation in mouse hepatocytes (Fig. 1D). Thus, we sought to determine if C10 could prevent hepatic TG accumulation *in vivo*. After 18 weeks of HF diet feeding and C10 treatment (Prevention Study), histological examination of the liver revealed the absence of steatosis in C10-treated mice when compared to livers of the HF sham and HF DMSO control mice. (Fig. 3A). To further quantify C10 inhibition of hepatic lipid accumulation, TG content was quantified biochemically which revealed increased TG content from liver samples of HF-fed mice when compared to LF-fed mice. Treatment with C10 reduced hepatic TG content compared to HF sham and HF DMSO groups (Fig. 3B). There was no difference noted among adipose depot weights of HF diet groups; however, the difference in hepatic TG content in the liver of C10-treated mice when compared to the HF controls indicate that C10 may have a localized effect on hepatic lipid metabolism. Serum TG and cholesterol levels were measured in non-fasted mice after 16 weeks of HF diet feeding (Fig. 4A and B, respectively). Total serum TG remained unchanged (Fig. 4A) while total serum cholesterol was elevated in HF diet-fed mice when compared to LF sham controls; C10 did not affect serum cholesterol levels in HF-fed mice (Fig. 4B).

### C10 protects C57BL/6J male mice from HF diet-induced glucose intolerance

**Figure 5:**
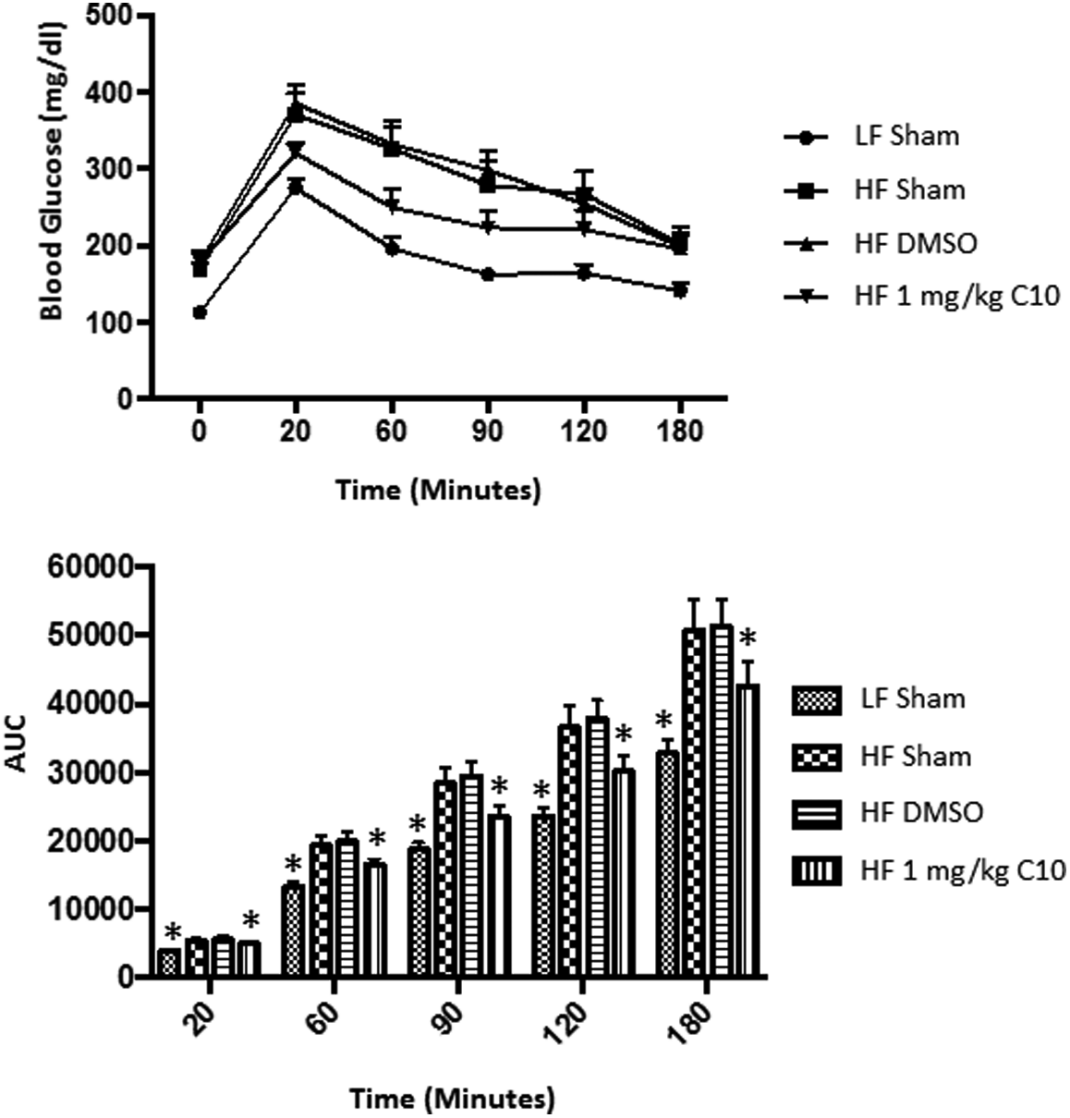
C10-treated mice maintain glucose tolerance despite obesity. A 3 h intraperitoneal glucose tolerance test was performed after 13 weeks of HF diet feeding. C10 prevented HF diet-induced glucose intolerance. Area under the curve was significantly lower in the LF sham group as well as the HF C10-treated group throughout the duration of the glucose challenge. Data points on the line graph indicate mean and error bars indicate +/− s.e.m. and bars on the bar graph indicate mean + s.e.m. Significance was determined using ANOVA followed by Tukey’s *post hoc* analysis for multiple comparison; *Different from HF sham and HF DMSO groups; *P* < 0.05, *n* = 8.

Ectopic fat deposition in insulin target tissues impairs the function of insulin signaling and thus impairs glucose homeostasis (i.e. induces glucose intolerance/insulin resistance). Previous *in vitro* studies have shown that treatment with C10 prevent palmitate-induced IRS1 serine 307 phosphorylation, a process known to mediate insulin resistance in insulin-stimulated 3T3L1 adipocytes (McCall *et al*. 2010). To determine the effect of C10 on glucose tolerance in HF diet-fed mice, we performed a 2-h intraperitoneal glucose tolerance test (IPGTT) 13 weeks post diet and treatment initiation. The HF-fed control animals exhibited glucose intolerance, with significantly higher glucose levels at 20, 60, 90, 120 and 180 min following glucose administration during the IPGTT compared to LF-fed mice (Fig. 5), and the area under the curve (AUC) was significantly higher in the HF-fed mice compared to LF-fed mice (Fig. 5). HF-fed mice receiving C10 treatment had improved glucose tolerance despite their obesity as compared to the HF sham and HF DMSO-treated mice (Fig. 5).

### C10 prevents HF diet-induced inflammation in liver and mesenteric adipose tissue from C57BL/6J male mice

**Figure 6:**
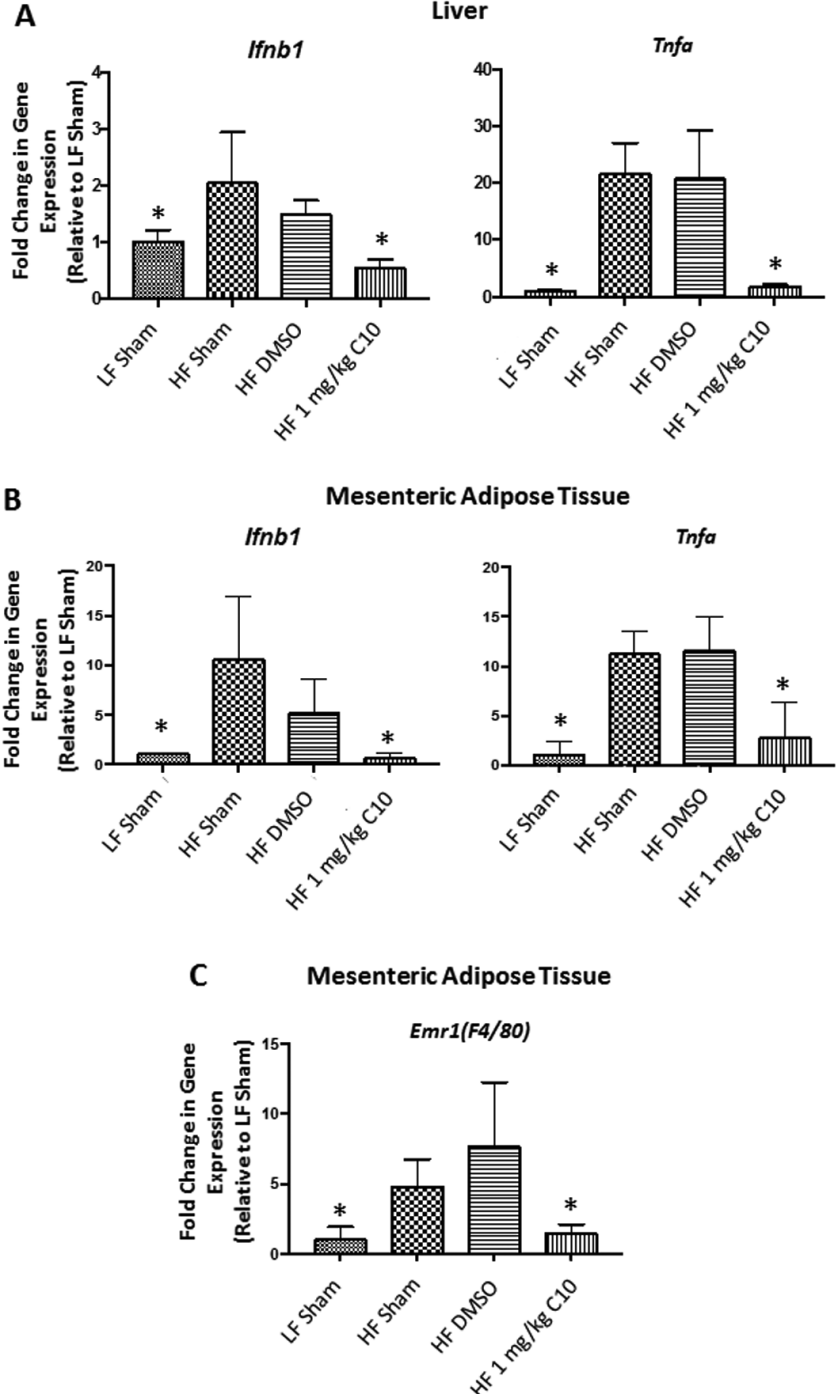
C10 prevents HF diet-induced inflammation *in vivo*. Inflammatory gene expression was measured in liver and mesenteric adipose tissue after 18 weeks of HF diet feeding. Hepatic *Ifnb1* and *Tnfa* expression were reduced in C10-treated mice when compared to HF sham and DMSO groups (A). Additionally, C10 prevented an upregulation of *Ifnb1* and *Tnfa* in mesenteric adipose tissue (B) as well as *Emr1*(*F4/80*), a macrophage marker (C). Bars indicate mean + s.e.m. Significance was determined using ANOVA followed by Tukey’s *post hoc* analysis for multiple comparison; *Different from HF sham and HF DMSO groups; *P* < 0.05, *n* *=* 7–8.

Inflammation, specifically due to cytokines and chemokines produced by FFA and gut-derived LPS activation of TLR4 signaling, leads to systemic glucose intolerance by impairing insulin signaling in target tissues including adipose and liver. Activation of the TLR4 signaling pathways leads to expression of pro-inflammatory cytokines, in particular, *Tnfa* and type 1 interferons (*Ifnb1*). Our model of HF diet feeding promotes an increase in circulating FFAs, gut-derived LPS, as well as an increase in fat deposition and accumulation in adipose tissue and the liver (Fraulob *et al*. 2010). Pathologic exposure of adipose and liver tissue to FFAs and gut-derived LPS activates TLR4 signaling and induces a local inflammatory tissue response marked by an increase in pro-inflammatory cytokine gene expression. We observed that palmitate treatment induces expression of *Tnfa* and *Ifnb1* in AML-12 cells, which was inhibited by treatment with C10 (Fig. 1). Additional studies in our laboratory have shown that C10 prevents transcriptional activity of NFKB and IRF3 thereby inhibiting the upregulation of cytokine and chemokine production (McCall *et al*. 2010, 2013, Deosarkar *et al*. 2014). To determine the efficacy of C10 to prevent HF diet-induced inflammation, adipose and liver tissue were collected from the mice in this study for analysis of pro-inflammatory gene expression. In both adipose and liver tissue, there was a significant increase in *Tnfa* and *Ifnb1* expression in our HF sham and HF DMSO groups (Fig. 6). However, as in our *in vitro* studies, this HF diet-mediated increase in *Tnfa* and *Ifnb1* mRNA levels was inhibited in both liver and adipose tissues from the HF diet-fed C10-treated animals (Fig. 6A and B, respectively). Additionally, mRNA levels of *F4/80*, which encodes a cell surface macrophage marker remained low in adipose tissue of C10-treated mice despite HF diet feeding (Fig. 6C).

### C10 reverses HF diet-induced glucose intolerance, hepatic and adipose inflammation and hepatic steatosis

**Figure 7:**
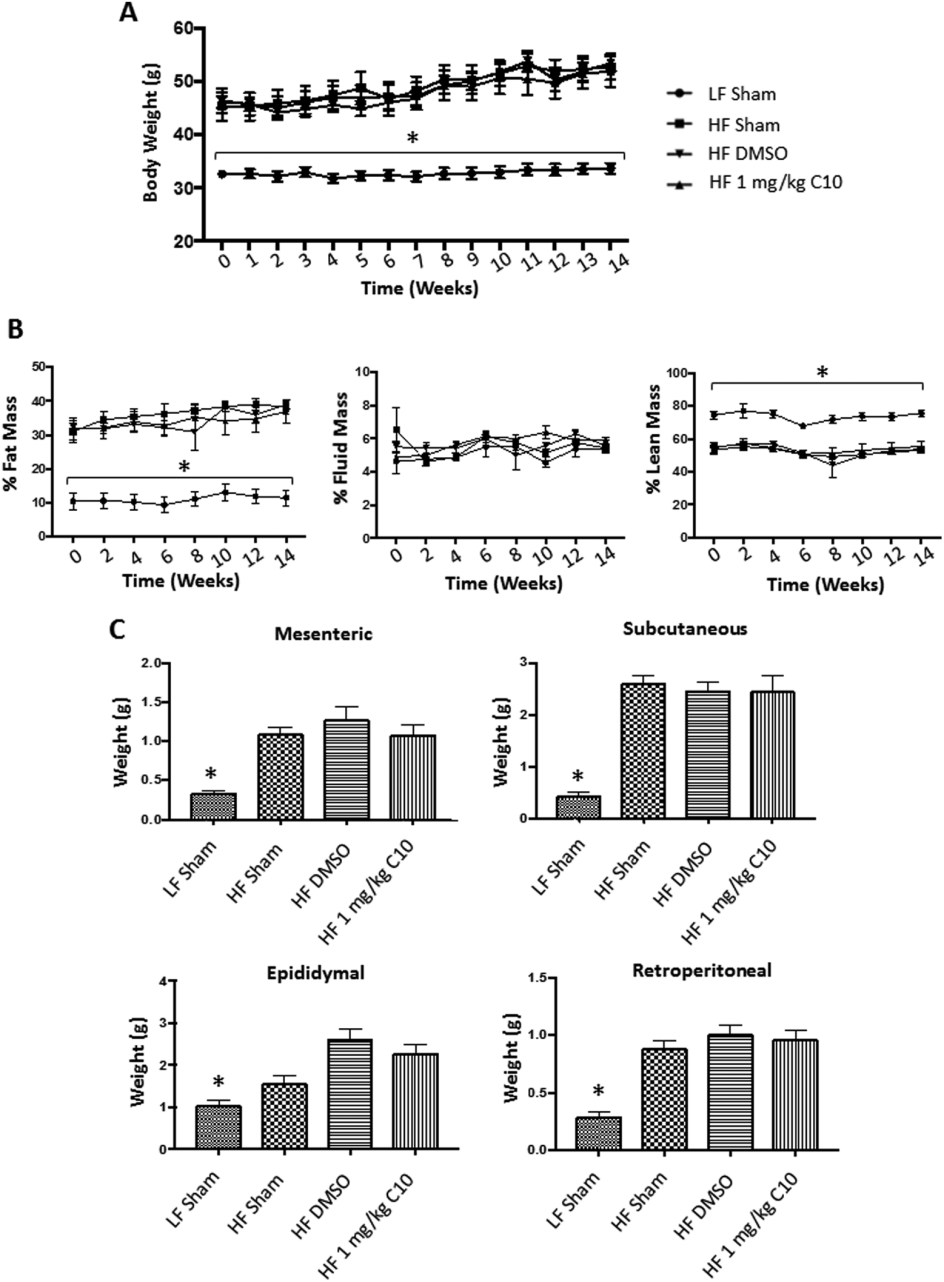
C10 did not reverse body weight increase due to HF diet feeding. Weeks 0–14 are represented as the start and end points of the C10 ‘reversal study’ post 16 weeks of HF or LF diet feeding. Total body weight was measured weekly and body composition was measured every 2 weeks during the duration of the study. At the end of the study, adipose tissue weight was measured. (A) Body weight of HF diet-fed mice remained significantly greater than LF diet-fed mice. (B) Additionally, body composition revealed that HF diet-fed mice had significantly greater % Fat Mass as well as a decrease in % Lean Mass when compared to the LF-fed mice. Percent Fluid Mass was no different between LF- and HF-fed mice. (C) Adipose (Subcutaneous, Mesenteric, Epididymal, and Retroperitoneal) tissue weight was greater in the HF diet-fed mice when compared to the LF diet-fed mice. C10 did not reverse the increase in adipose tissue mass due to HF diet feeding. Data points on line graphs (A and B) indicate mean and error bars indicate +/− s.e.m. and bars on bar graphs (C) indicate mean + s.e.m. Significance was determined using ANOVA followed by Tukey’s *post hoc* analysis for multiple comparison; *Different from HF-fed groups; *P* < 0.05, *n* = 5–6.

**Figure 8:**
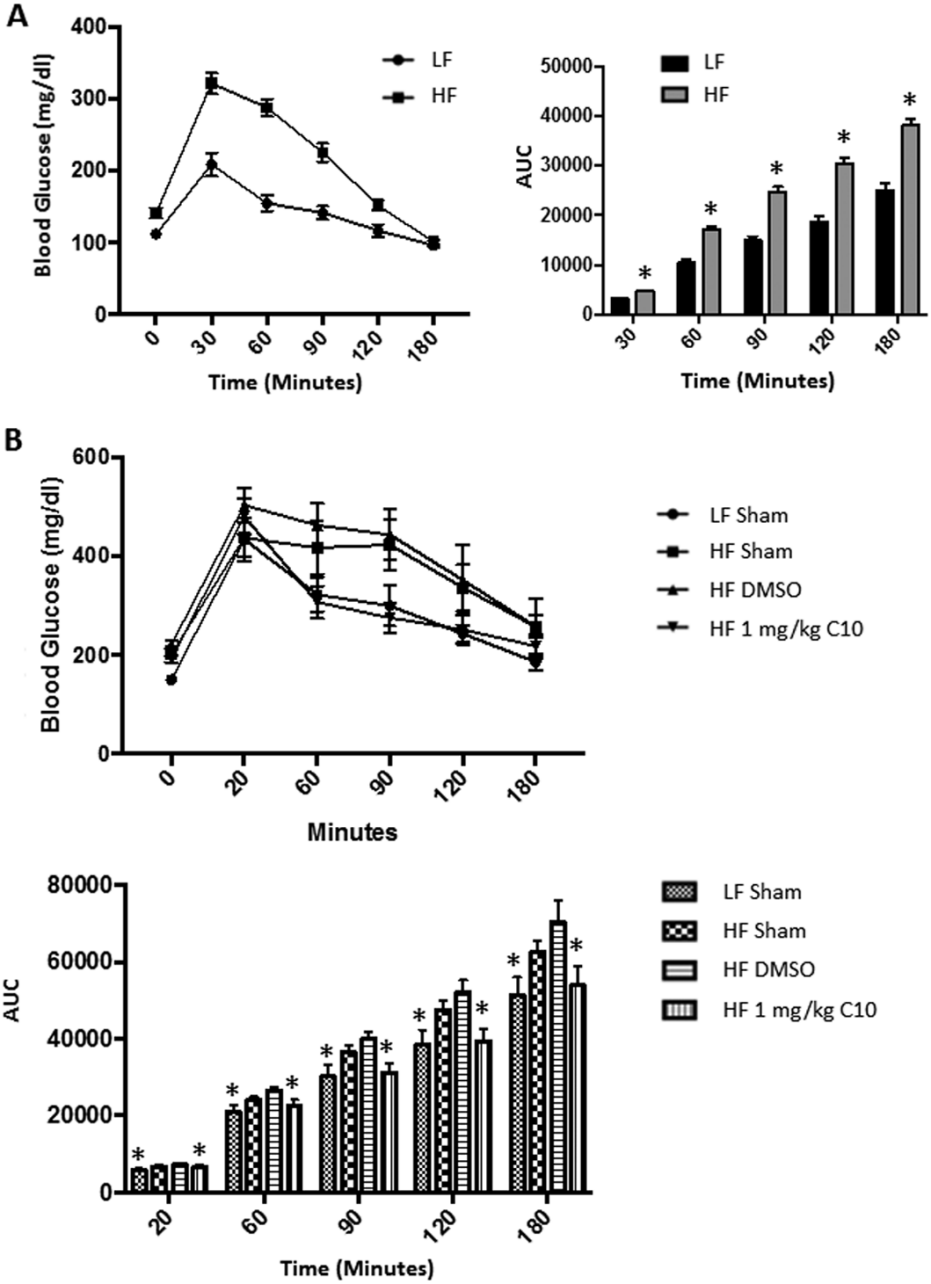
C10 treatment reverses HF diet-induced glucose intolerance. A 3 h intraperitoneal glucose tolerance test was performed just prior to the initiation of treatment (A) and after 12 weeks from the start date of the reversal study (B). (A) At the beginning of the study all HF-fed mice were glucose intolerant compared to LF-fed mice. (B) Following treatment, blood glucose levels remained elevated in the HF sham and HF DMSO groups when compared to the LF sham and HF C10-treated mice. Area under the curve was significantly lower in the C10-treated group when compared to the HF sham and DMSO groups and was nearly indistinguishable from the LF sham group. This indicates that C10 reverses glucose intolerance due to the HF diet feeding. Data points on line graphs indicate mean and error bars indicate +/− s.e.m. and bars on bar graphs indicate mean + s.e.m. Significance was determined using ANOVA followed by Tukey’s *post hoc* analysis for multiple comparison; (A) * Different from LF sham, (B) * Different from HF sham and DMSO groups; *P* < 0.05, *n* = 5–6.

**Figure 9:**
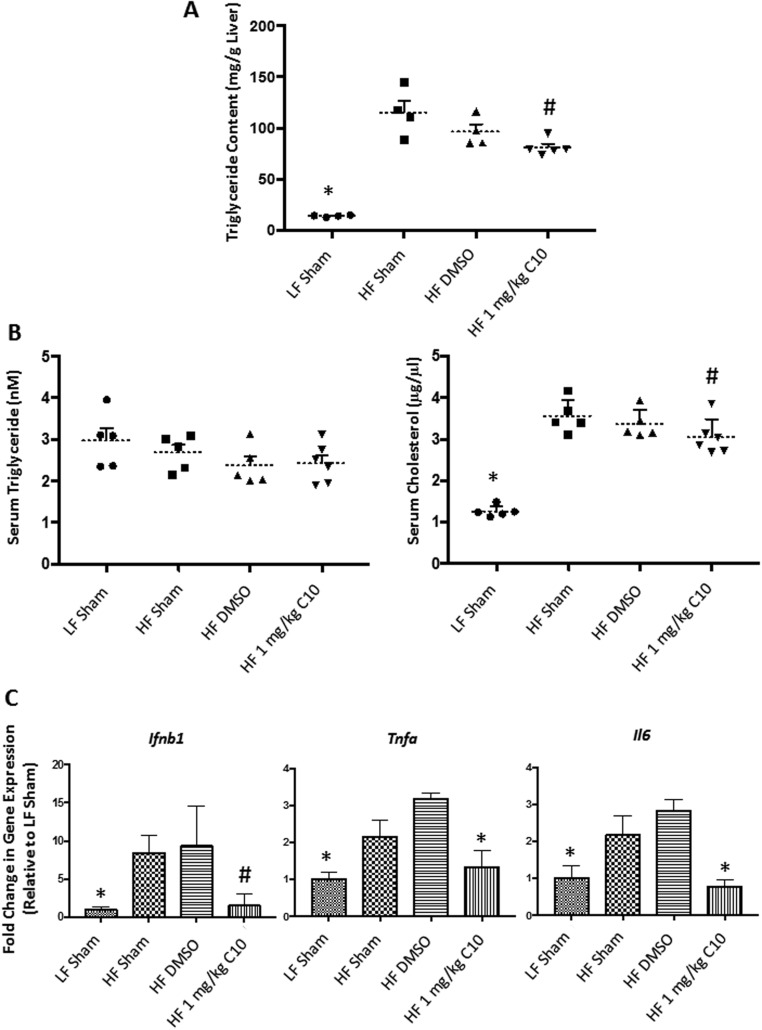
C10 treatment reverses HF diet-induced hepatic steatosis and hepatic and adipose inflammation and reduces serum cholesterol. (A) Treatment with C10 decreased hepatic triglyceride content when compared to HF-fed sham and DMSO groups. (B) Total serum cholesterol levels were reduced in the HF-fed C10-treated mice when compared to HF-fed control mice, however, serum triglyceride levels were unchanged. Dotted lines represent the mean and error bars indicate + s.e.m. (C) Hepatic *Ifnb1*, *Tnfa* and *Il6* expression was reduced in C10-treated mice when compared to HF sham and DMSO groups. Bars indicate mean + s.e.m. Significance was determined using ANOVA followed by Tukey’s *post hoc* analysis for multiple comparison; ^#^Different from HF sham and DMSO groups; *P* < 0.05. *Different from HF-fed groups;* P* < 0.05,* n* = 4–6.

The observation that C10 prevents HF diet-induced hepatic steatosis and inflammation as well as adipose inflammation and glucose intolerance in our DIO mouse model led us to question if C10 could reverse established glucose intolerance, hepatic steatosis and hepatic inflammation in these mice. To address this, male C57BL/6J mice were put on either a LF diet or a HF diet for 16 weeks after which glucose tolerance was evaluated in each mouse via IPGTT. Mice on the LF diet that were glucose tolerant remained in the study and were maintained on the LF diet for the duration of the experiment. Mice on the HF diet that were glucose intolerant remained in the study and were maintained on the HF diet for the remainder of the study. As can be seen in Figs 7 and 8, the LF-fed mice weighed significantly less than the HF-fed mice (Fig. 7A, Week 0) and the IPGTT revealed that the LF-fed mice were glucose tolerant, whereas the HF-fed mice were glucose intolerant (Fig. 8A). One week later (after 17 weeks on the diets), the glucose-intolerant HF-fed mice were randomly divided into HF sham, HF DMSO and HF C10 (1 mg/kg) treatment groups as described earlier for the ‘prevention study’ and were treated as indicated for 14 weeks. There was no difference in weights between the HF-fed treatment groups (Fig. 7A), although all HF-fed groups continued to gain weight and become more obese over the course of the 14-week treatment period (Fig. 7); however, the HF-fed C10-treated mice were now glucose tolerant (Fig. 8B), indicating that despite a continued rise in obesity, C10 reversed the glucose intolerance that was present in the mice prior to C10 treatment (Fig. 8A and B). Hepatic steatosis (Fig. 9A) was also reduced as well as total serum cholesterol (Fig. 9B). Serum TG remained unchanged (Fig. 9B). Moreover, hepatic inflammation (*Tnfa*, *Ifnb1* and *Il6*) (Fig. 9C) was dramatically reduced in C10-treated mice compared to the HF controls.

## Discussion

Inflammation is widely recognized as a key factor in the pathogenesis of metabolic diseases, specifically obesity-related diseases such as T2DM and NAFLD. Obesity alone is considered the most important risk factor for development of NAFLD and is the driver of inflammation in this disease that is responsible for its progression (Wild *et al*. 2004, Dabelea *et al*. 2014, Imes & Burke 2014). The major inflammatory signaling pathway in chronic inflammation in a state of obesity is TLR4 (Davis *et al*. 2008, Pierre *et al*. 2013, Jia *et al*. 2014, Sawada *et al*. 2014). TLR4 is abundantly expressed in insulin target tissues such as adipose tissue, liver and skeletal muscle and is now accepted as a key player in obesity-induced insulin resistance and T2DM (Jialal *et al*. 2014). Earlier studies suggested that the stimulation of TLR4 seen in obesity/insulin resistance/T2DM results from gut-derived LPS (Lassenius *et al*. 2011, Jayashree *et al*. 2014, Velloso *et al*. 2015); however, it is now evident that FFAs derived from HF diets can also trigger TLR4 signaling in these target tissues (Reyna *et al*. 2008, Kim *et al*. 2012) leading to NAFLD and insulin resistance.

In our model, we used a HF diet to promote the development of insulin resistance and hepatic steatosis. HF diets increase circulating levels of FFAs, which deposit in adipose tissue and other tissues such as the liver. Acceleration of hepatic FFA deposition occurs in obesity-induced NAFLD due to an increase in dietary fatty acids or lipolysis of adipose tissue. We hypothesized that C57BL/6J mice fed a HF diet would develop hepatic inflammation, steatosis and insulin resistance, which would be prevented and/or reversed with C10 treatment. Human and mouse hepatocyte cell lines demonstrated an inflammatory response when exposed to LPS and FFAs. In our *in vitro* system, C10 exhibited potent anti-inflammatory properties by preventing FFA- and LPS-induced pro-inflammatory cytokine expression measured by a reduction in *Tnfa* and *Ifnb1* mRNA levels in hepatocytes in culture. Similar findings were observed *in vivo* as pro-inflammatory cytokine expression was also significantly reduced by C10 in liver and mesenteric adipose tissue of mice fed a HF diet. In addition to anti-inflammatory effects, C10 treatment prevented glucose intolerance (an indirect measure of insulin resistance) and hepatic steatosis in mice fed a HF diet. Inhibition of FFA-induced hepatic lipid accumulation by C10 treatment was also observed *in vitro.* Furthermore, and most clinically relevant, HF diet-induced insulin resistance was reversed by C10 intervention. C10-treated mice also had significantly reduced hepatic inflammation and decreased hepatic TG content, albeit the latter effect was not as dramatic as was observed in the ‘prevention study’. The modest effect of C10 on hepatic TG content in the ‘reversal study’ may be due to the fact that the HF diet used in this study induced an overwhelming amount of hepatic steatosis due to the HF diet containing 60% fat. If the C10 treatment had continued for a longer duration or the diet changed to regular chow, we anticipate this effect would be more pronounced, especially given the fact that hepatic inflammation and insulin resistance was significantly reduced. In this regard, we have previously shown that continued HF feeding after intensive insulin therapy, in this same mouse model, prevents the ‘Legacy Effect’ of early insulin treatment in new-onset T2DM (Guo *et al*. 2015). While not evaluated in this study, it would be of interest in future studies to see if other models of NAFLD (e.g. *ob/ob* or *db/db* mice) also respond similarly to C10 treatment.

Currently, there are no therapeutic interventions to prevent the inflammation associated with NAFLD. Because NAFLD is associated with metabolic disease and is often considered the hepatic manifestation of metabolic syndrome, pharmacological agents that target the lipid accumulation or insulin resistance component of NAFLD are used as front-line therapies. Certain anti-diabetic therapies including pioglitazone (Ratziu *et al*. 2008), acarbose (Chiasson *et al*. 2002), metformin (Haukeland *et al*. 2009) and possibly statins (Eslami *et al*. 2013) exhibit anti-inflammatory properties and are effective at treating NAFLD. However, there is a real need for novel, new classes of anti-inflammatory drugs for the prevention and treatment of the localized inflammation associated with NAFLD as the inflammation in the presence of steatosis is what leads to NASH and the more severe stages of the disease that result in death.

The pathogenesis of NAFLD is now considered to be ‘multiple-hit’ due to hepatic insults that occur in parallel, which ultimately leads to increased lipid accumulation and immune infiltration (Day & James 1998). The early stage of NAFLD (steatosis) is considered benign; however, it is now believed to be an active state of inflammation and metabolic dysfunction (Tilg & Moschen 2010, Buzzetti *et al*. 2016). Moreover, we now know that inflammation occurs in hepatic steatosis and thus it can be targeted by pharmacological agents before the onset of NASH. Activation of TLR4 signaling is a key mediator of HF diet-induced hepatic inflammation.

In addition to being a critical mediator of TLR4 signaling, MyD88 has recently been shown to be critical for maintaining mammalian target of rapamycin (mTOR) activation (Chang *et al*. 2013). Given the metabolic consequences of NAFLD (i.e. obesity, insulin resistance and T2DM), mTOR involvement is essential in light of its role in cardiovascular diseases such as atherosclerosis, coronary heart disease and stroke (Tarantino & Capone 2013, Patil & Sood 2017). Moreover, a key mechanism linking inflammation to altered glucose and lipid metabolism is that visceral adipocytes and associated macrophages produce and release copious amounts of inflammatory cytokines into both the portal and systemic vasculature, which cause insulin resistance in insulin target tissues (i.e. liver, muscle and fat). Thus, the novel findings presented herein that C10 can reverse HF diet-induced hepatic steatosis, glucose intolerance, as well as hepatic and visceral adipose inflammation, coupled with the finding that C10 inhibits *Tnfa*-induced *Vcam1* expression and reduces monocytic cell adhesion to endothelial cells (Dagia *et al*. 2004), an important process in the pathogenesis of atherosclerosis and other chronic inflammatory diseases, suggests that C10 may have a more profound clinical impact than the treatment of NAFLD alone.

## Declaration of interest

K D M, D J G and F S are inventors on a patent issued to Ohio University on the subject matter.

## Funding

This work was supported in part by the JO Watson Endowment for Diabetes Research and 2020 Vision grant from the Ohio University Heritage College of Osteopathic Medicine, the Ohio University Heritage College of Osteopathic Medicine McCall Research Support Fund, and the John J. Kopchick Molecular and Cellular Biology (MCB)/Translational Biomedical Sciences (TBS) Fellowship Award to A P.

## Author contribution statement

A P, T C, C W, J T, D G, D E B, E O L, F S, K M wrote the manuscript. A P, T C, C W, J T, D E B, E O L, F S, and K M designed and performed research and analyzed data. K M contributed new reagents/analytical tools.
